# Long-term clinical outcomes in females with precocious puberty treated with gonadotropin-releasing hormone analogues

**DOI:** 10.3389/fendo.2026.1911743

**Published:** 2026-10-05

**Authors:** Nahla Khawaja, Khalid Karaki, Mohammad K. Ruba’i, Yousef Khader, Arwa Kinani, Oraib Farahid, Mohammed El-Khateeb, Omar Ali, Abdulrahman Abujbara, Laith Hyasat, Kamel Ajlouni

**Affiliations:** 1 Department of Endocrinology, The National Center (Institute) for Diabetes, Endocrinology, and Genetics, The University of Jordan, Amman, Jordan; 2 Department of Public Health, Jordan University of Science and Technology (JUST), Irbid, Jordan; 3 Faculty of Medicine, The University of Jordan, Amman, Jordan

**Keywords:** gonadotropin-releasing hormone analogues, insulin resistance, long term outcomes, PCOS (polycystic ovarian syndrome, precocious puberty

## Abstract

**Objective:**

To evaluate long-term anthropometric, metabolic, reproductive, and bone mineral density (BMD) outcomes in girls with central precocious puberty (CPP) treated with Gonadotropin-Releasing Hormone analogues (GnRHa).

**Methods:**

In this retrospective cohort with cross-sectional follow-up assessment, 82 of 265 eligible females (30.9%) with central precocious puberty or central hypothalamic-pituitary-gonadal axis activation who had received GnRHa participated in long-term evaluation. Anthropometric outcomes, insulin sensitivity indices (HOMA-IR and QUICKI), hormonal profile, BMD, and reproductive outcomes including polycystic ovary syndrome (PCOS) were assessed. Analyses used available cases for each outcome; missing values were not imputed.

**Results:**

The mean age at final evaluation was 17.61 ± 4.3 years, and mean GnRHa treatment duration was 3.14 ± 1.9 years. Mean height at final follow-up was 159.85 ± 6.5 cm; the mean difference from baseline predicted adult height was 1.98 cm (95% CI, -0.37 to 4.34; P = 0.097). The distribution of BMI categories did not change significantly from baseline to final follow-up. Mean HOMA-IR and QUICKI were 3.76 ± 1.82 and 0.319 ± 0.025, respectively; among adults, 14/28 (50.0%) met each prespecified adult-derived threshold for reduced insulin sensitivity. PCOS was identified in 15/67 participants (22.4%). Mean lumbar and femoral BMD Z-scores were -0.25 ± 1.23 and -0.55 ± 1.03, respectively.

**Conclusions:**

Among participants who completed long-term follow-up, attained height was comparable at the group level with predicted adult and mid-parental height, and mean BMD Z-scores were within age- and sex-referenced ranges. PCOS and PCOS-related features were observed, while insulin sensitivity indices were associated with adiposity. Because only 30.9% of eligible patients participated and there was no untreated comparison group, these findings describe the evaluated cohort and should not be interpreted as causal effects of GnRHa treatment.

## Introduction

Central precocious puberty (CPP) is one of the common disorders in pediatric endocrinology, caused by premature activation of hypothalamic pituitary gonadal (HPG) axis. It is characterized by the appearance of secondary sexual characteristics before the age of 8 years in girls and 9 years in boys (1). Gonadotropin-releasing hormone analogues (GnRHa) constitute the mainstay treatment for CPP. Therapy aims to suppress premature activation of the hypothalamic pituitary gonadal axis, thereby preserving predicted adult height and achieving age-appropriate pubertal milestones, while also considering the child’s psychosocial well-being and coping capacity (2).

Longstanding widespread use of GnRHa for the treatment of idiopathic CPP clearly demonstrated their efficacy in restoring genetic growth potential and improving final adult height particularly if treatment is initiated at a younger age (3). However, long-term effects have not been fully elucidated and increasing attention has been directed toward the long-term metabolic, skeletal, and reproductive consequences of GnRHa exposure.

Long-term reproductive outcomes are one of the major concerns for the treating physicians and the families simultaneously. Studies evaluating pubertal recovery, fertility and reproduction potential are reassuring (4–7). Persistent uncertainties regarding the risk of polycystic ovary syndrome (PCOS) are underscored in several studies (5, 6). CPP and PCOS are interrelated reproductive and metabolic disorders at different life stages with shared pathophysiological and genetic features, it is speculated that both conditions could have the same underlying neuroendocrine dysfunction in the form of altered gonadotropin dynamics and represent the two ends of the same spectrum (8). The role of GnRHa therapy in modulating subsequent PCOS risk remains controversial.

Puberty is a critical window for bone mass accrual and consolidation, as 40% of peak bone mass is acquired between Tanner stages II and V (9). Compromised bone health is another concern related GnRHa therapy as gonadal steroid suppression may lead to deceleration of skeletal maturation and temporary decline in bone mineral density acquisition (4). However, the detrimental effects are not long lasting, as bone mineral density (BMD) typically return to baseline after discontinuation of treatment, with no clear adverse effect on peak bone mass (10). Nevertheless, long-term follow-up assessment studies evaluating BMD at final adult height and beyond are limited (4, 5).

Precocious puberty is commonly associated with obesity and reduced insulin sensitivity as baseline adiposity will enhance early activation of the hypothalamic–pituitary–gonadal axis (11, 12). This baseline adverse metabolic profile will influence their long-term cardiometabolic risk (13). The impact of GnRHa on body mass index (BMI), BMI standard deviation scores (BMI-SDS) and insulin sensitivity during and after treatment remains controversial (12, 14). Moreover, several concerns were raised whether the observed metabolic changes reflect treatment effects or related to their underlying predisposition.

Although GnRHa therapy is well established for preserving height potential in girls with idiopathic CPP, uncertainty remains regarding its long-term metabolic, reproductive, and skeletal consequences, particularly in populations with high background rates of overweight and insulin resistance (4–6, 10). Data from Jordan have shown favorable height outcomes after treatment, but long-term information on PCOS, insulin sensitivity, and bone health is lacking (15, 16). Therefore, this study aimed to evaluate long-term anthropometric, metabolic, reproductive, and bone mineral density outcomes in girls with idiopathic CPP treated with GnRHa, and to explore their associations with treatment duration and baseline clinical characteristics.

## Methods

### Study design and participants

This study was conducted as a retrospective cohort with cross-sectional follow-up assessment at the National Center for Diabetes, Endocrinology, and Genetics (NCDEG), Amman, Jordan. This institution is a tertiary referral center for pediatric and adult endocrinology and serves patients from across Jordan. The study included girls diagnosed with idiopathic CPP or central pubertal activation who received treatment with GnRHa between January 2003 and December 2022. Eligible participants were females who had been diagnosed and treated for idiopathic central precocious puberty or central pubertal activation and received intramuscular GnRHa therapy (triptorelin, Ipsen Pharma) at a dose of 3.75 mg administered every 28 days for a minimum duration of one year. Among patients treated during the study period, 265 met the eligibility criteria for long-term follow-up. Patients were subsequently recalled for follow-up evaluation at least two years after menarche to assess long-term anthropometric, metabolic, reproductive, and skeletal outcomes. The final cohort consisted of 82 participants who were successfully contacted, agreed to participate, and completed the long-term follow-up assessment. Of these, 71 had documented breast development consistent with the conventional clinical definition of idiopathic CPP, whereas 11 had Tanner breast stage 1 documented at the initial clinical assessment despite biochemical evidence of central HPG-axis activation and advanced skeletal maturation that had led the treating endocrinologist to initiate GnRHa therapy according to historical institutional practice.

Exclusion criteria included the presence of organic causes of precocious puberty, such as central nervous system lesions, history of cranial trauma, or prior cranial irradiation. Patients were also excluded if they had endocrine disorders associated with hyperandrogenism as congenital adrenal hyperplasia or other conditions that can cause menstrual irregularity, including hypothyroidism and hyperprolactinemia. Participants using medications that could influence metabolic or hormonal parameters—such as hormonal contraception, anti-androgen therapy, insulin sensitizers, or systemic glucocorticoids—within six months prior to the evaluation were also excluded. Patients with incomplete clinical records or those who declined participation in the follow-up assessment were not included in the final analysis.

### Diagnosis of idiopathic central precocious puberty and central HPG-axis activation

The diagnosis of CPP had been established at the time of presentation according to the institutional standards during the study period and it was based on integration of clinical, auxological, radiological, and biochemical findings. Clinical evidence of precocious puberty required early pubertal development, particularly the onset of breast development before 8 years of age or menarche before the age of 10 years, accompanied by bone age advancement by at least 1 year beyond chronological age and central HPG-axis activation confirmed by a pubertal luteinizing hormone (LH) response following GnRH stimulation testing. A stimulated LH level exceeding the assay-specific pubertal cutoff, which ranged from approximately 3.3 to 5.0 IU/L depending on the assay methodology and institutional diagnostic protocols applied at that time, was considered evidence of HPG-axis activation (2). Stimulated LH results were interpreted together with age at pubertal onset, clinical pubertal progression, growth velocity, and degree of bone-age advancement rather than in isolation.

GnRH/GnRHa stimulation protocols and LH assay platforms changed during the study period according to institutional practice. From 2003 to 2010, stimulation testing was performed using 100 µg of intravenous gonadorelin (HRF). From 2010 to 2022, testing was performed using 0.1 mg of triptorelin (Decapeptyl). Measurements were performed using different assays according to laboratory availability and changes in institutional practice over an extended period from 2003 to 2022. In 2003, measurements were performed using the Abbott IMx microparticle enzyme immunoassay (MEIA). The Abbott AxSYM MEIA platform was used from 2004 to 2005, followed by the Abbott Architect i1000 from 2005 to 2011 and the Architect i2000 from 2011 to 2016, both of which used chemiluminescent microparticle immunoassay technology. From 2016 to 2018, LH was measured using the Siemens ADVIA Centaur XPT direct chemiluminometric immunoassay, followed by the Mindray CL-900i chemiluminescent immunoassay platform from 2018 to 2022.

### GnRHa treatment initiation

Among participants meeting the diagnostic criteria for idiopathic CPP, GnRHa treatment was initiated following individualized assessment by the treating endocrinologist. Institutional clinical practice for initiating GnRHa in ICPP patients during the study period are clinical evidence of precocious pubertal development, bone-age advancement of at least one year beyond chronological age, and a pubertal LH response following GnRH/GnRHa stimulation testing. Factors considered by the treating endocrinologist included age at pubertal onset, Tanner-stage progression, growth velocity, degree of bone-age advancement, predicted adult height, biochemical evidence of central HPG-axis activation and psychosocial considerations. During the study period, no predefined numerical cut-off for predicted adult height or uniform definition of rapidly progressive puberty was used to determine treatment eligibility. Because this study retrospectively covered a 20-year period, treatment decisions reflected routine clinical practice and were individualized according to the overall assessment of clinical, auxological, radiological, and hormonal profile.

### GnRHa monitoring and discontinuation

All participants received intramuscular triptorelin 3.75 mg every 28 days. Treatment response was monitored clinically by evaluating pubertal stage, growth velocity, bone-age progression, and hormonal assessment when clinically indicated. Treatment duration and discontinuation were individualized rather than based on uniform predefined thresholds throughout the study period. The decision to discontinue GnRHa therapy was individualized by the treating endocrinologist based on chronological age, bone age, pubertal stage, growth velocity, remaining growth potential, and psychosocial considerations.

Adherence to GnRHa therapy was evaluated using available medical records and documented GnRHa injection dates. Information regarding delayed or missed GnRHa injections was not consistently documented for all participants. Growth hormone or other height-promoting therapies were not administered to any participant in this study.

### Data collection and clinical evaluation

Clinical and demographic data were obtained through systematic review of medical records at the time of CPP diagnosis and during routine endocrinology clinic follow-up visits, and by direct clinical evaluation at the time of the long-term follow-up assessment. Age at pubertal onset was defined as the age at which breast development or other secondary sexual characteristics were first observed by the family. This information was obtained from medical records; however, it was not consistently documented for all participants. Age at diagnosis was defined as the age at the first endocrine evaluation at which CPP was confirmed using clinical, radiological, and biochemical criteria. Age at initiation of GnRHa therapy was defined as the age at administration of the first GnRHa injection. Treatment was generally initiated at the time of diagnosis or shortly thereafter; however, the exact diagnosis-to-treatment interval was not consistently documented and could not be reliably determined for all participants. Data extracted from medical records included baseline anthropometric measurements (height and BMI), mid-parental height (MPH), bone age (BA), predicted adult height (PAH), age at initiation and discontinuation of GnRHa therapy, duration of treatment, and the interval between treatment cessation and menarche. Bone age was assessed using the Greulich and Pyle atlas method, predicted adult height (PAH) was calculated using the Bayley–Pinneau prediction tables adjusted for bone age advancement and MPH for girls was calculated as the average of parental height minus 6.5cm.

At the time of follow-up evaluation, all participants underwent a standardized clinical assessment performed by trained clinicians according to a predefined protocol. Anthropometric measurements included height and weight, which were measured without shoes and with participants wearing light clothing using a calibrated stadiometer and digital scale. Participants were evaluated two years after menarche, a time at which the majority of linear growth had occurred. BMI was calculated as weight in kilograms divided by the square of height in meters (kg/m²) and expressed as age- and sex-adjusted standard deviation scores (BMI-SDS).

For participants aged ≥18 years at final follow-up, BMI was classified according to standard adult categories as normal weight (<25 kg/m²), overweight (25.0–29.9 kg/m²), and obesity (≥30 kg/m²). For participants younger than 18 years, BMI percentiles were used to classify weight status according to standard pediatric criteria:

- Normal weight: BMI <85th percentile
- Overweight: BMI ≥85th to <95th percentile
- Obesity: BMI ≥95th percentile.

Menstrual and reproductive history were obtained using a structured interview, including age at menarche, menstrual cycle regularity, and interval between treatment discontinuation and the onset of spontaneous menarche. Clinical hyperandrogenism was assessed through systematic clinical examination, including evaluation for acne and hirsutism. Hirsutism was quantified using the modified Ferriman–Gallwey (mFG) scoring system, with a score ≥8 considered indicative of clinically significant hirsutism. Fasting venous blood samples were collected in the morning after an overnight fast during the early follicular phase of the menstrual cycle (days 2–4). Serum concentrations of follicle-stimulating hormone (FSH), luteinizing hormone (LH), total testosterone (TT), free testosterone ((FT), dehydroepiandrosterone sulfate (DHEAS), and 17-hydroxyprogesterone (17-OHP) were measured. To exclude secondary causes of hyperandrogenism, serum prolactin, thyroid-stimulating hormone (TSH), and free thyroxine (free T4) were also measured. Participants with basal 17-hydroxyprogesterone levels ≥6 nmol/L underwent adrenocorticotropic hormone (ACTH) stimulation testing to exclude non-classical congenital adrenal hyperplasia.

Fasting plasma glucose and insulin levels were measured to evaluate metabolic status. Insulin resistance and insulin sensitivity were assessed using two validated indices: the Homeostasis Model Assessment of Insulin Resistance (HOMA-IR) and the Quantitative Insulin Sensitivity Check Index (QUICKI). HOMA-IR was calculated using the formula:

HOMA-IR = fasting insulin (µU/mL) × fasting glucose (mmol/L)/22.5, with values ≥1.984 considered indicative of insulin resistance among (17). Insulin sensitivity was also evaluated using QUICKI, calculated as: QUICKI = 1/[log (fasting insulin, µU/mL) + log (fasting glucose, mg/dL)], with values ≤ 0.344 indicating reduced insulin sensitivity (17). Because the prespecified thresholds of HOMA-IR ≥1.984 and QUICKI ≤0.344 were derived from an adult population, these cutoffs were applied only to participants aged ≥18 years.

Transabdominal pelvic ultrasonography was performed to assess ovarian morphology using a GE Healthcare high-resolution ultrasound system (LOGIQ S8) equipped with C1–6 convex transabdominal transducer (GE Healthcare, Chicago, IL, USA). All examinations were performed by an experienced radiologist following standardized pelvic imaging protocols. Ovarian dimensions and follicular characteristics were recorded. Because transabdominal ultrasonography does not permit reliable assessment of the total follicle number throughout the ovary, total follicle count was not used to define PCOM. Polycystic ovarian morphology was defined as an ovarian volume ≥10 mL in at least one ovary (20).

Bone mineral density (BMD) was measured using Dual-Energy X-ray Absorptiometry (DEXA) with a GE Lunar iDXA system. Measurements were obtained at the lumbar spine and femoral neck, and BMD values were expressed as grams per square centimeter (g/cm²) and age- and sex-adjusted Z-scores. Low bone mineral density was defined as a Z-score ≤ −2.0, in accordance with the International Society of Clinical Densitometry (ISCD) guidelines (18).

### Diagnosis of polycystic ovary syndrome and hormonal assays

PCOS was diagnosed according to the 2023 International Evidence-Based Guideline for the Assessment and Management of Polycystic Ovary Syndrome and the international adolescent-specific recommendations (19, 20). The diagnostic criteria were applied according to gynecological age rather than chronological age alone. Gynecological age was defined as the interval between age at menarche and age at assessment for PCOS. In participants with gynecological age ≥ 8 years, PCOS was diagnosed based on the 2023 International Evidence-Based Guideline for the Assessment and Management of Polycystic Ovary Syndrome, which requires the presence of at least two of the following three features:

1. Oligo-ovulation or anovulation,
2. Clinical and/or biochemical hyperandrogenism, and
3. Polycystic ovarian morphology (PCOM) on pelvic ultrasound (19).

In participants within 8 years of menarche, PCOS was diagnosed according to the international adolescent-specific recommendations, which require the presence of both persistent menstrual irregularity and clinical and/or biochemical hyperandrogenism, while pelvic ultrasonographic evidence of PCOM was not used as a diagnostic criterion in participants within 8 years of menarche (20). Participants presenting with only one of these two features were classified as being at risk of PCOS rather than having definite PCOS. Participants who did not fulfill the criteria for PCOS or at-risk status were classified as non-PCOS.

Persistent menstrual irregularity was defined according to the number of years since menarche, as menstrual cycles shorter than 21 days or longer than 45 days between 2 and <3 years after menarche. From ≥3 years after menarche, cycles shorter than 21 days or longer than 35 days, or fewer than eight menstrual cycles per year, were considered abnormal (19, 20). Clinical hyperandrogenism was defined as the presence of moderate to severe acne and/or hirsutism assessed by the modified Ferriman–Gallwey score.

Biochemical hyperandrogenism was defined as serum androgen concentrations exceeding the age- and assay-specific upper reference limit for at least one androgen, including TT, FT, or DHEAS.

Serum free testosterone (FT) and 17-OHP concentrations were measured using MAGLUMI chemiluminescent immunoassay (CLIA) technology (Snibe Diagnostic, Shenzhen, China). The analytical sensitivity of the assays was ≤0.100 pg/mL for free testosterone and ≤0.212 nmol/L for 17-OHP. The intra-assay coefficients of variation (CV) were 3.8% and 3.84%, respectively, and the inter-assay CV were 3.01% and 1.65%. The reference ranges for females in the follicular phase were <4.2 pg/mL for FT and 0.30–2.42 nmol/L for 17-OHP. Serum total testosterone (TT) and DHEAS were measured using chemiluminescent immunoassay (CLIA) on the CL-8000i automated immunoassay analyzer (Mindray, Shenzhen, China). The analytical sensitivity was <0.025 ng/mL for total testosterone and ≤2.0 μg/dL for DHEAS. The intra-assay CV were 1.48% and 2.51%, respectively, while the inter-assay CV were 3.86% and 5.17%. The reference ranges for females in the follicular phase were <1.23 ng/mL for total testosterone and 63–360 μg/dL for DHEAS.

Serum follicle-stimulating hormone (FSH), luteinizing hormone (LH), insulin, prolactin, thyroid-stimulating hormone (TSH), and free thyroxine (free T4) were also measured using chemiluminescent immunoassay on the CL-8000i analyzer (Mindray, Shenzhen, China) according to the manufacturer’s standardized protocols.

All laboratory analyses were performed in the accredited clinical laboratory of the National Center for Diabetes, Endocrinology, and Genetics using standardized quality-control procedures.

### Statistical analysis

Statistical analyses were performed using IBM SPSS Statistics, version 28.0 (IBM Corp., Armonk, NY, USA). Continuous variables are presented as mean ± SD or median (IQR), depending on their distribution, and categorical variables as counts and percentages. Normality was evaluated using the Shapiro-Wilk test together with visual inspection of histograms and Q-Q plots, and statistical tests were selected accordingly. Analyses were conducted using available cases for each outcome, without imputation of missing values. Outcome-specific denominators are reported in the text and tables; paired BMI data were available for 78 participants, HOMA-IR/QUICKI for 73, PCOS assessment for 67, and BMD for 77.

Changes in BMI categories between baseline and final follow-up were assessed using the McNemar-Bowker test. For comparative analyses, participants were categorized according to the presence or absence of definite PCOS. The ‘no definite PCOS’ group included participants classified as either at risk of PCOS or non-PCOS. Continuous variables were compared using the independent-samples t test or Mann-Whitney U test, as appropriate, and categorical variables using Pearson’s chi-square test or Fisher’s exact test. For binary comparisons reconstructed from summary counts, Fisher’s exact test was used when an expected cell count was below 5 or an observed cell was zero; otherwise, Pearson’s chi-square test was used. Because menstrual irregularity, clinical or biochemical hyperandrogenism, and PCOM contributed directly to PCOS classification, their P values are provided only for descriptive completeness and are not interpreted as independent associations. Correlations between insulin sensitivity indices and anthropometric measures or treatment duration were examined using Pearson correlation analysis.

Multivariable linear regression was used to examine factors associated with height at final follow-up. Because age at diagnosis and bone age at presentation were collinear, they were entered into separate models. Both models included predicted adult height at diagnosis and duration of GnRHa treatment; one model included age at diagnosis and the other bone age at presentation. Results are reported as regression coefficients with 95% CIs and represent associations rather than causal treatment effects. A multivariable PCOS model was not retained because only 15 definite PCOS cases were available, which was insufficient for a stable model containing the proposed predictors. All tests were two-sided, and P < 0.05 was considered statistically significant. Analyses were exploratory; no adjustment for multiple testing was applied, and P values should be interpreted cautiously. A sensitivity analysis excluded the 11 participants with breast Tanner stage 1 documented at initial presentation and repeated the principal descriptive outcomes in the remaining 71 participants with the conventional clinical CPP phenotype.

## Results

### Patients’ characteristics

A total of 427 patients diagnosed with CPP and treated with GnRHa between 2003 and 2022 were identified. Of these, 265 met the eligibility criteria. Among eligible patients, 145 (54.7%) could not be reached and 38 (14.3%) declined participation; therefore, 82/265 (30.9%) completed the long-term assessment and 183/265 (69.1%) did not participate (**Figure 1**). The mean age at diagnosis/GnRHa initiation was 7.78 ± 1.5 years, mean bone age was 10.25 ± 1.4 years, and mean bone-age advancement was 2.24 ± 0.9 years. Mean baseline predicted adult height was 157.86 ± 10.4 cm. At presentation, breast enlargement was documented in 71 (86.6%) participants, pubic hair in 63 (76.8%), and axillary hair in 61 (74.4%); 12 (14.6%) had experienced menarche before GnRHa initiation. Baseline characteristics are summarized in **Table 1**. Mean GnRHa treatment duration was 3.14 ± 1.9 years. At final evaluation, mean age was 17.61 ± 4.3 years, 48 participants were younger than 18 years, and mean time since treatment discontinuation was 6.60 ± 3.8 years (range, 3–19 years; **Table 2**).

**Figure 1:**
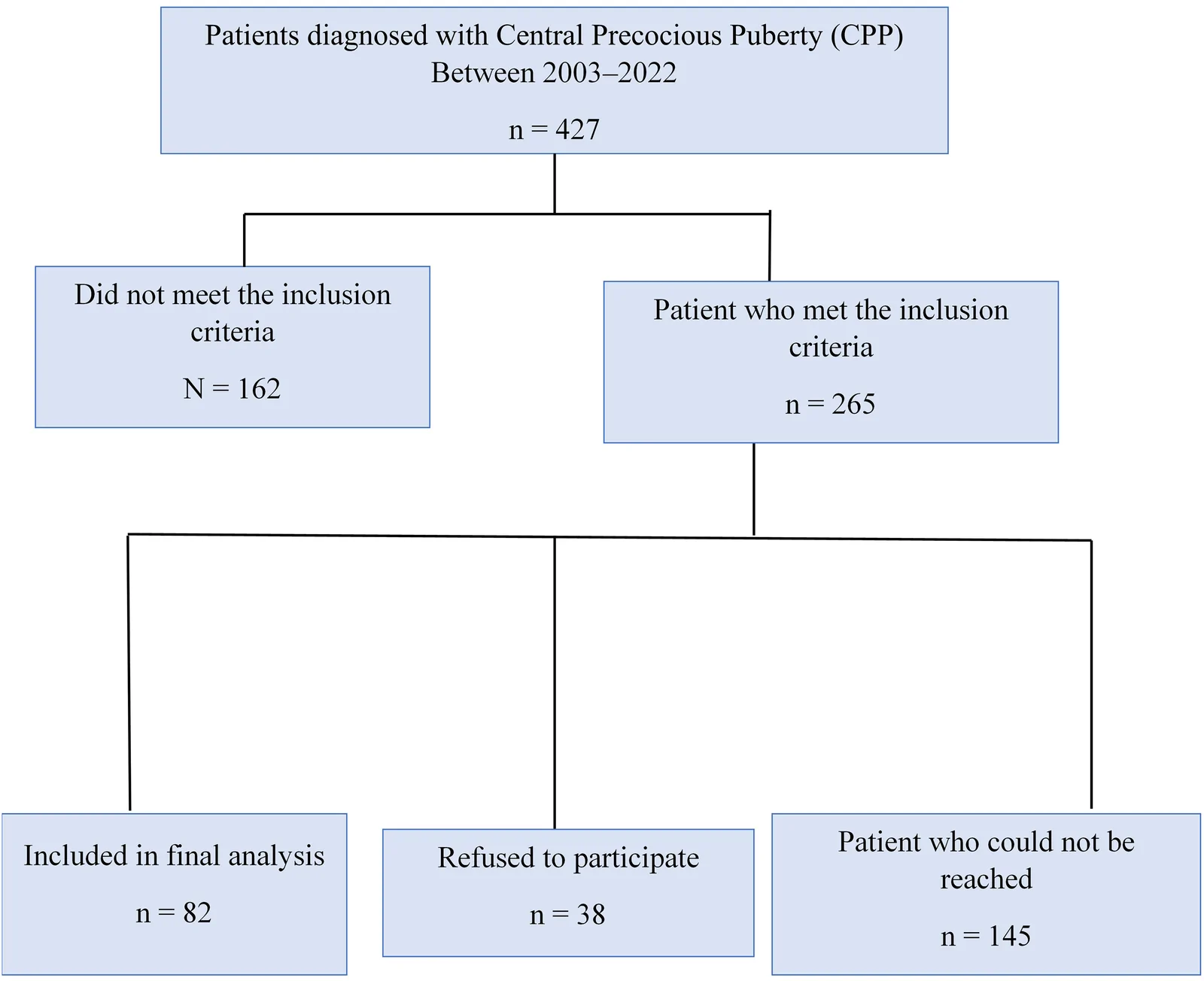
Flow diagram of study population and final sample selection.

**Table 1:** Baseline clinical, auxological, and pubertal characteristics of study participants (n = 82).

Demographic and auxological characteristics	Mean ± SD
Age at diagnosis/GnRHa initiation, years	7.78 ± 1.5
Bone age at diagnosis, years	10.25 ± 1.4
Bone-age advancement, years	2.24 ± 0.9
Height at diagnosis, cm	132.82 ± 12.0
Mid-parental height, cm	159.99 ± 5.5
Predicted adult height at diagnosis, cm	157.86 ± 10.4
BMI at diagnosis, kg/m²	19.62 ± 3.4
BMI SDS at diagnosis	1.09 ± 0.91
Pubertal characteristics at presentation	
Breast enlargement, n (%)	71 (86.6)
Pubic hair present, n (%)	63 (76.8)
Axillary hair present, n (%)	61 (74.4)
Menarche before GnRHa initiation, n (%)	12 (14.6)
*Breast Tanner stage documented at initial presentation, n (%)	
Tanner stage 1	11 (13.4)
Tanner stage 2	35 (42.7)
Tanner stage 3	13 (15.9)
Tanner stage 4	6 (7.3)
# Not recorded	17 (20.7)

*Tanner stage refers to the breast stage documented at the initial clinical evaluation and may not correspond to the stage at the time of GnRHa initiation.

#Breast enlargement was documented in the medical record but Tanner stage was not recorded.

GnRHa, Gonadotropin-Releasing hormone analogue, BMI, Body mass index, BMI SDS, Body mass index standard deviation score.

**Table 2:** Treatment and follow-up outcomes characteristics of study participants (n = 82).

Treatment characteristics	Mean ± SD
Age at treatment discontinuation, years	11.63 ± 1.3
Duration of GnRHa treatment, years	3.14 ± 1.9
Follow-up characteristics	Mean ± SD
Age at final follow-up, years	17.61 ± 4.3
Time since treatment discontinuation, years	6.60 ± 3.8
Auxological characteristics at follow-up	Mean ± SD
Height at end of treatment, cm	150.53 ± 6.6
Final follow-up height, cm	159.85 ± 6.5
Final follow-up height - Mid-parental height	0.10 ± 5.62
Final follow-up height - baseline PAH, cm	1.98 ± 10.1
BMI at end of treatment, kg/m²	22.83 ± 4.2
BMI SDS at end of treatment	1.04 ± 0.78
BMI at final follow-up, kg/m²	25.89 ± 5.9
BMI SDS at final follow-up	0.86 ± 1.16

In the subgroup who had experienced menarche before initiation of GnRHa therapy, the mean chronological age at diagnosis/GnRHa initiation was 8.54 ± 0.71 years and the mean bone age was 11.23 ± 1.44 years. The mean height at GnRHa initiation was 138.09 ± 6.44 cm. The mean duration of GnRHa treatment in this subgroup was 1.91 ± 0.80 years.

Among the 11 participants with breast Tanner stage 1 documented at the initial clinical evaluation, all had biochemical evidence of central HPG-axis activation on GnRH/GnRHa stimulation testing together with advanced skeletal maturation. Mean age at GnRHa initiation was 8.38 ± 0.96 years and mean bone age was 10.8 ± 1.4 years. Nine participants had documented pubic and/or axillary hair at presentation, while three had experienced menarche before treatment initiation. GnRHa therapy was started at the initial presentation in four participants, after approximately 6 months in three, and after approximately 12 months in two; the interval from presentation to treatment initiation could not be reliably determined for the remaining two participants.

### Long-term outcomes

#### Height at final follow-up

At diagnosis of precocious puberty, the mean height was 132.82 ± 12.0 cm and the PAH was 157.86 ± 10.4 cm. The mean height at final follow-up was 159.85 ± 6.5 cm. The mean difference between height at final follow-up and MPH was 0.10 cm (SD 5.62; 95% CI, −1.21 to 1.41; P = 0.883). The mean difference between height at final follow-up and PAH was 1.98 cm (SD 10.16; 95% CI, −0.37 to 4.34; P = 0.097). At final evaluation, 74.3% of participants achieved a height with in the predefined target height of their PAH at diagnosis, which was defined as ± 5 cm (± 1 SD from the predicted value). For the 12 participants who had experienced menarche before GnRHa initiation, the mean height at final follow-up was 154.08 ± 5.22 cm. and the difference between height at final follow-up and baseline PAH was 0.1 ± 5.2 cm.

Multivariable linear regression analyses were performed to identify factors associated with height at final follow-up (**Table 3**). Two separate models were constructed to avoid collinearity: Model 1 included age at diagnosis, predicted adult height at diagnosis, and duration of GnRHa treatment, while Model 2 included bone age at presentation, predicted adult height at diagnosis, and duration of GnRHa treatment. In both models, higher PAH at diagnosis and longer duration of GnRHa treatment were independently associated with greater height at final follow-up as shown in **Table 3**. While, later age at diagnosis (β = −1.061, 95% CI: −2.061 to −0.061; P = 0.038) and more advanced bone age at presentation (β = −0.950, 95% CI: −1.89 to −0.007; P = 0.048) were associated with lower height at final follow-up in Model 1 and Model 2, respectively.

**Table 3:** Multivariable linear regression analysis of factors associated with height at final follow-up.

Variable	Model 1 β	95% CI	P value	Model 2 β	95% CI	P value
Predicted adult height at diagnosis	0.263	0.137 - 0.388	<0.001	0.309	0.159 -0.459	<0.001
Duration of GnRHa treatment	1.313	0.396 - 2.229	0.006	1.676	0.645 - 2.706	0.002
Age at diagnosis	-1.061	−2.061 – (-0.061)	0.038	—	—	—
Bone age at presentation	—	—	—	-0.950	−1.89 – (-0.007)	0.048

• Model 1 includes age at diagnosis, predicted adult height at diagnosis and duration of GnRHa treatment.

• Model 2 includes bone age at presentation, predicted adult height at diagnosis and duration of GnRHa treatment

• β values represent change in height at final follow-up (cm) per 1-unit increase in the predictor.

• CI = 95% confidence interval.

### BMI

The mean BMI SDS at diagnosis was 1.09 ± 0.91. According to BMI percentiles, 52.6%, 15.4% and 32.1% were classified as normal weight, overweight and obese at initiation of GnRHa, respectively. At the conclusion of treatment, the mean BMI SDS was 1.04± 0.78. According to BMI percentiles, 60.4%, 14.6% and 25% were classified as normal weight, overweight and obese, respectively. At final evaluation, the mean BMI SDS was 0.86 ± 1.16. According to BMI percentiles, 45.1%, 25.6% and 29.3% were classified as normal weight, overweight and obese, respectively. Paired analyses were performed to evaluate changes in BMI SDS over time. BMI SDS did not change significantly during treatment, with a mean BMI SDS of 1.09 ± 0.91 before treatment and 1.05 ± 0.78 at the conclusion of therapy (P = 0.989). In contrast, the mean BMI SDS decreased significantly after treatment completion, from 1.03 ± 0.79 at the end of treatment to 0.66 ± 1.38 at final evaluation (P = 0.021). Over the entire follow-up period, mean BMI SDS decreased from 0.97 ± 0.91 before treatment to 0.86 ± 1.17 at final evaluation but this change was not statistically significant (P = 0.410).

The transitions in BMI categories from baseline to the current assessment are shown in **Table 4**. A total of 78 participants were included in the paired BMI analysis. Among participants with normal BMI at diagnosis, 73.2% remained normal, while 26.8% shifted to higher BMI categories. Patients who were overweight at baseline demonstrated a marked shift toward higher BMI categories as 58.3% became obese, 25.0% remained overweight, and 16.7% transitioned to the normal weight category. Of those classified as obese at baseline, 44% improved to a lower BMI category, whereas 56% remained obese. Despite these individual transitions, the overall distribution of BMI categories did not change significantly over time, as demonstrated by the McNemar–Bowker test of symmetry (χ² = 4.05, df = 3, P = 0.256).

**Table 4:** Transition of BMI categories from baseline to final assessment (n=78).

*Baseline BMI category	BMI Category at final follow-up		
	Normal	Overweight	Obese
Normal (n=41)	30 (73.2%)	8 (19.5%)	3 (7.3%)
Overweight (n=12)	2 (16.7%)	3 (25.0%)	7 (58.3%)
Obese (n=25)	2 (8.0%)	9 (36.0%)	14 (56.0%)
Total (n=78)	34	20	24

Data are presented as number (row percentage).

Statistical test: McNemar–Bowker test of symmetry (χ² = 4.05, df = 3, P = 0.256)

*BMI categories were defined according to age- and sex-specific percentiles recorded in the study dataset: For participants aged < 18 years at final follow-up, normal weight, BMI <85th percentile; overweight, BMI ≥85th to <95th percentile; and obesity, BMI ≥95th percentile. For participants aged ≥18 years at final follow-up, BMI was classified according to standard adult categories as normal weight (<25 kg/m²), overweight (25.0–29.9 kg/m²), and obesity (≥30 kg/m²). Because the final follow-up included both adolescents and adults, longitudinal category transitions should be interpreted cautiously.

### Insulin resistance

At recruitment for the 73 participants with available data, the mean fasting blood glucose was 94.11 ± 7.46 mg/dl and the mean fasting insulin level was 16.39 ± 7.52 µIU/ml. The mean HOMA-IR was 3.76 ± 1.82 (median 3.42; range, 0.99–8.99), while the mean QUICKI was 0.319 ± 0.025 (median 0.318; range, 0.28–0.38). Among participants aged ≥18 years, the mean HOMA-IR was 3.09 ± 1.61 (median 2.59; range, 0.99–6.48), and the mean QUICKI was 0.331 ± 0.026 (median 0.327; range, 0.29–0.38). Among participants aged <18 years, the mean HOMA-IR was 4.20 ± 1.84 (median 4.02; range, 1.76–8.99), while the mean QUICKI was 0.312 ± 0.018 (median 0.310; range, 0.28–0.35). Mean HOMA-IR increased across BMI categories at final follow-up as it was found to be 2.89 ± 1.21, 3.90 ± 1.61 and 5.00 ± 2.05 in normal, overweight and obese patients, respectively (P=<0.001). Because the prespecified HOMA-IR and QUICKI thresholds were derived from an adult population, categorical classification was restricted to participants aged ≥18 years. Among participants ≥18 years, (n = 28), 14 participants (50%) had a HOMA-IR ≥1.984 and 14 (50%) had a QUICKI ≤0.344 based on the prespecified adult-derived thresholds. Pearson correlation analysis demonstrated significant positive correlations between BMI, BMI SDS and HOMA-IR across different assessment periods as shown in **Table 5**. The strongest association was observed between BMI-SDS at final follow-up and HOMA-IR (r = 0.590, P < 0.001). On the other hand, QUICKI index showed significant negative correlations with BMI and BMI SDS across different assessment periods. The strongest inverse association was observed with BMI-SDS before treatment (r = -0.358, P = 0.002) and BMI at final follow-up (r = −0.416, P < 0.001). No statistically significant correlation was found between HOMA-IR (r = −0.202, p = 0.089), QUICKI index (r = 0.199, P = 0.093) and duration of GnRHa therapy.

**Table 5:** Correlation of anthropometric parameters and treatment duration with insulin resistance and sensitivity indices (HOMA-IR and QUICKI) at final follow-up (n=73).

Variable	HOMA-IR		QUICKI index	
	Pearson r	P value	Pearson r	P value
BMI before treatment	0.524	<0.001	-0.342	0.003
BMI SDS before treatment	0.450	<0.001	-0.358	0.002
BMI at final follow-up	0.493	<0.001	-0.416	<0.001
BMI SDS at final follow-up	0.590	<0.001	-0.244	0.039
BMI at the end of treatment	0.433	0.004	-0.272	0.078
BMI SDS at the end of treatment	0.472	0.002	-0.315	0.042
Duration of GnRHa therapy	-0.202	0.089	0.199	0.093

Pearson correlation test was used. Statistical significance was set at P < 0.05.

### Reproductive outcomes

The age at menarche was 12.65 ± 1.4 years. Spontaneous menarche occurred 1.08 ± 0.9 years after discontinuation of treatment with a range of 3 to 72 months. At final follow up, the mean gynecological age of the cohort was 6.18 ± 3.84 years. Regular menstrual cycles were reported in 79.1% of the study population, 62.1% were having acne and 46.3% were having hirsutism. At final follow up, three participants reported spontaneous pregnancies without the use of assisted reproductive technologies, including *in vitro* fertilization (IVF).

Polycystic ovary syndrome was assessed in 67 of 82 participants: 15 (22.4%) met criteria for definite PCOS, 27 (40.3%) were classified as at risk of PCOS, and 25 (37.3%) were classified as non-PCOS. Among 49 participants evaluated using adolescent-specific criteria because they were within 8 years after menarche, 8 (16.3%) met criteria for definite PCOS. Among 18 participants with gynecological age ≥ 8 years evaluated using adult criteria, 7 (38.9%) met diagnostic criteria. Of these seven, two had hyperandrogenism, ovulatory dysfunction, and PCOM; one had hyperandrogenism and ovulatory dysfunction without PCOM; and four had hyperandrogenism and PCOM without ovulatory dysfunction.

Participants with definite PCOS (n = 15) were compared with those without definite PCOS (n = 52; at-risk and non-PCOS categories combined). For diagnostic components, P values reconstructed from the reported counts are shown in **Table 6** for descriptive completeness. Because these variables contributed to PCOS classification, the observed differences are expected by construction and are not interpreted as independent associations.

**Table 6:** Clinical, biochemical and metabolic characteristics according to polycystic ovary syndrome (PCOS) status at final follow-up (n=67).

Outcome	Total (N = 67)	PCOS group n = 15 (22.4%)	≠ No definite PCOS group n = 52 (77.6%)	P value
Reproductive characteristics				
Age at menarche (years)	12.65 ± 1.48	12.43 ± 1.26	12.71 ± 1.54	0.523
Duration of GnRHa treatment (years)	3.12 ± 1.72	3.10 ± 1.32	3.13 ± 1.83	0.968
Interval from treatment discontinuation to menarche (years)	0.97 ± 0.68	0.72 ± 0.43 (0.2–4)	1.04 ± 0.73 (0.2–3)	0.112
Gynecological age at PCOS assessment, years	6.18 ± 3.84 (2–19)	7.42 ± 3.36 (2–12)	5.79 ± 3.93 (2-19)	0.185
Anthropometric measures				
BMI SDS at diagnosis	1.16 ± 0.86	1.08 ± 0.59	1.18 ± 0.93	0.690
BMI at final follow-up (kg/m²)	26.51 ± 5.71	28.18 ± 7.95	26.03 ± 4.87	0.201
BMI SDS at final follow-up	0.93 ± 1.25	1.41 ± 0.52	0.87 ± 1.30	0.417
Obesity at baseline n/N (%)	22/67 (32.8%)	6/15 (40.0%)	16/52 (30.8%)	0.283
Menstrual pattern				
Regular menstrual cycles n/N (%)	53/67 (79.1)	4/15 (26.7)	49/52 (94.2)	<0.001†
Irregular menses n/N (%)	14/67 (20.9)	11/15 (73.3)	3/52 (5.8)	–
Clinical hyperandrogenism				
Acne n/N (%)	41/66 (62.1)	15/15 (100)	26/51 (51.0)	0.001†
Hirsutism n/N (%)	31/67 (46.3)	11/15 (73.3)	20/52 (38.5)	0.017†
Acne and hirsutism n/N (%)	28/67 (41.8)	11/15 (73.3)	17/52 (32.7)	0.005†
Acne or hirsutism n/N (%)	44/67 (65.7)	15/15 (100)	29/52 (55.8)	0.001†
Metabolic parameters				
HOMA-IR (n=67)	3.83 ± 1.86	3.39 ± 1.91	3.94 ± 1.84	0.327
QUICKI (n=67)	0.32 ± 0.02	0.32 ± 0.03	0.31 ± 0.02	0.121
*Insulin resistance, n/N (%)	14/28 (50)	5/9 (55.6)	9/19 (47.4)	0.686
Hormonal profile (n=67)				
LH (mIU/mL)	6.12 ± 3.90	7.59 ± 4.71	5.69 ± 3.57	0.608
FSH (mIU/mL)	6.71 ± 2.56	6.41 ± 1.19	6.80 ± 2.84	0.224
LH/FSH ratio	0.98 ± 0.6	1.15± 0.7	0.93 ± 0.6	0.913
LH/FSH ≥ 2 n/N (%)	4/66 (6.1)	1/15 (6.7)	3/51 (5.9)	1.000
Total testosterone (ng/mL)	0.50 ± 0.34	0.65 ± 0.41	0.45 ± 0.30	0.051
Free testosterone (pg/mL)	2.10 ± 0.96	2.18 ± 0.86	2.07 ± 1.0	0.711
17-OH progesterone (nmol/L)	2.96 ± 1.53	3.43 ± 1.61	2.82 ± 1.50	0.192
DHEA-S (µg/dL)	226.81 ± 124.89	248.09 ± 109.4	221.49 ± 128.88	0.496
Prolactin (ng/mL)	20.82 ± 10.22	24.70 ± 11.78	19.68 ± 9.55	0.095
TSH (µIU/mL)	2.50 ± 1.35	3.04 ± 1.61	2.34 ± 1.24	0.086
Free T4 (pmol/L)	14.93 ± 2.18	14.67 ± 2.05	15.00 ± 2.22	0.622
Biochemical hyperandrogenism n/N (%)	6/67 (9.0)	3/15 (20.0)	3/52 (5.8)	0.121†
**Ultrasound findings				
PCOM absent n/N (%)	49/66 (74.2)	8/15 (53.3)	41/51 (80.4)	0.048†
PCOM present n/N (%)	17/66 (25.8)	7/15 (46.7)	10/51 (19.6)	–

Data are presented as mean ± SD or n/N (%). Percentages are calculated using the number of participants with available data for each variable. Because of missing data, the effective sample size may vary across variables; the corresponding sample size (n or N) is indicated for each parameter where applicable.

≠The “no definite PCOS” group included participants classified as at risk of PCOS (n = 27) and those classified as non-PCOS (n = 25)

*Insulin resistance was classified only among adult participants (N = 28) using the prespecified adult-derived HOMA-IR threshold of ≥1.984 (17).

**PCOM: Polycystic ovaries morphology using transabdominal ultrasound examination defined as an ovarian volume ≥10 mL in at least one ovary. In participants within 8 years of menarche, PCOM was reported descriptively and was not used as a diagnostic criterion for PCOS.

†P values were reconstructed from the reported 2 × 2 counts using Fisher’s exact test when an expected cell count was below 5 or an observed cell was zero, and Pearson’s chi-square test otherwise. Menstrual irregularity, clinical/biochemical hyperandrogenism, and PCOM contributed to PCOS classification; therefore, these P values are provided for descriptive completeness and are not interpreted as independent associations.

Among participants with definite PCOS, 11/15 (73.3%) had irregular menses, 15/15 (100%) had acne or hirsutism, 3/15 (20.0%) had biochemical hyperandrogenism, and 7/15 (46.7%) had PCOM. Corresponding frequencies in the no-definite-PCOS group were 3/52 (5.8%), 29/52 (55.8%), 3/52 (5.8%), and 10/51 (19.6%), respectively. Summary-count comparisons yielded P < 0.001, P = 0.001, P = 0.121, and P = 0.048, respectively. These P values are reported for completeness but are not interpreted as independent evidence because the variables contributed to PCOS classification.

Menstrual pattern, the presence of either acne or hirsutism, and ultrasonographic PCOM differed significantly between groups. Patients with PCOS were more likely to have menstrual irregularities in comparison to no definite PCOS group (73.3% vs. 5.8%, P < 0.001). Similarly, PCOM was present in 46.7% of patients with PCOS compared with 19.6% of those without definite PCOS (P = 0.035). The presence of acne and hirsutism was a common finding among patients with PCOS and significantly more frequent compared with the no definite PCOS group (73.3% vs. 32.7%, P = 0.005).

There were no significant differences between patients with PCOS and without definite PCOS regarding age at menarche (12.43 ± 1.26 vs. 12.71 ± 1.54 years, P = 0.523), duration of GnRHa treatment (3.10 ± 1.32 vs. 3.13 ± 1.83 years, p = 0.968), or the interval from treatment cessation to menarche (0.72 ± 0.43 vs. 1.04 ± 0.73 years, p = 0.112). Similarly, BMI and BMI SDS at final evaluation were comparable between the two groups.

HOMA-IR, QUICKI, LH, FSH, free testosterone, 17-hydroxyprogesterone, DHEA-S, prolactin, and thyroid function tests did not differ statistically between the definite-PCOS and no-definite-PCOS groups in these exploratory comparisons. Mean total testosterone was 0.65 ± 0.41 ng/mL in the definite-PCOS group and 0.45 ± 0.30 ng/mL in the no-definite-PCOS group (P = 0.051). Biochemical hyperandrogenism was present in 3/15 (20.0%) and 3/52 (5.8%), respectively (Fisher’s exact P = 0.121); this comparison is reported for descriptive completeness because biochemical hyperandrogenism contributed to PCOS classification (**Table 6**).

Among participants within 8 years of menarche, stratification by BMI category showed that PCOS was identified in 5 of 20 participants with normal BMI (25.0%), 1 of 15 participants with overweight (6.7%), and 2 of 14 participants with obesity (14.3%). Among participants with gynecological age ≥ 8 years, stratification by BMI category showed that PCOS was identified in 4 of 8 participants with obesity (50.0%), 2 of 5 participants with normal BMI (40.0%), and 1 of 5 participants with overweight (20.0%).

### Bone mineral density

Bone mineral density was evaluated in 77 of 82 participants. Mean lumbar spine BMD was 1.06 ± 0.18 g/cm² (mean Z-score, -0.25 ± 1.23), and mean femoral BMD was 0.94 ± 0.12 g/cm² (mean Z-score, -0.55 ± 1.03). The mean Z-scores were within the age- and sex-referenced range; however, these cross-sectional measurements do not show change from pretreatment values or establish an effect of GnRHa. BMD values and Z-scores across BMI categories are presented in **Table 7** as exploratory descriptive comparisons.

**Table 7:** Bone mineral density (BMD) and Z-scores according to body mass index (BMI) categories at final follow-up (n=77).

BMI Category	N	Lumbar BMD (g/cm²) Mean ± SD	Lumbar Z-score Mean ± SD	Femoral BMD (g/cm²) Mean ± SD	Femoral Z-score Mean ± SD
Total	**77**	1.06 ± 0.18	-0.25 ± 1.23	0.94 ± 0.12	-0.55 ± 1.03
Normal weight	36	1.02 ± 0.18	-0.77 ± 0.86	0.88 ± 0.10	-1.05 ± 0.74
Overweight	20	1.04 ± 0.22	0.10 ± 1.34	0.97 ± 0.11	-0.13 ± 1.03
Obese	21	1.14 ± 0.17	0.27 ± 1.37	1.01 ± 0.12	-0.10 ± 1.05
P value		0.017	0.003	<0.001	<0.001

BMI, body mass index; OR, odds ratio; CI, confidence interval; P-value, probability value. Normal BMI was used as the reference category for current BMI.

### Sensitivity analysis restricted to the conventional CPP phenotype

After exclusion of the 11 participants with breast Tanner stage 1 documented at initial presentation, 71 participants remained in the conventional CPP cohort phenotype. The main findings were essentially unchanged. Mean height at final follow-up was 159.99 ± 5.43 cm compared with 159.85 ± 6.50 cm in the full cohort, and the mean difference between height at final follow-up and PAH remained 1.98 cm. Similarly, mean HOMA-IR was 3.80 ± 1.87 compared with 3.76 ± 1.82 in the full cohort, while mean QUICKI was 0.319 ± 0.024 compared with 0.319 ± 0.025. PCOS was identified in 13 of 57 participants (22.8%) in the conventional CPP cohort compared with 15 of 67 (22.4%) in the full cohort. These findings indicate that exclusion of participants with breast Tanner stage 1 did not meaningfully affect the main auxological, metabolic, or reproductive outcomes (**Supplementary Table S1**).

## Discussion

This study represents a retrospective cohort of 82 girls with idiopathic CPP treated with GnRHa, followed by a cross-sectional comprehensive clinical evaluation several years after treatment discontinuation. Our analysis focused on height at final follow-up, BMI progression, insulin resistance, bone mineral density (BMD), pubertal recovery and polycystic ovary syndrome (PCOS). By integrating anthropometric, clinical, biochemical, bone mineral density and ultrasonographic data, we were trying to contribute to the ongoing debate regarding the long-term metabolic, skeletal, and reproductive outcomes in adulthood among females with idiopathic CPP following GnRHa therapy.

GnRHa have been extensively used primarily to restore genetic growth potential that is compromised by premature exposure to gonadal steroids. Height at final follow-up achieved in our cohort was 159.85 cm and was generally consistent with PAH and genetic target height, although individual responses varied substantially. Paired analyses showed that height at final follow-up did not differ significantly from MPH, with a mean individual difference of 0.10 cm (95% CI, −1.21 to 1.41; P = 0.883). Height gain can be used as a measure of treatment response to GnRHa therapy. In our cohort, the mean difference between height at final follow-up and baseline PAH was 1.98 cm; however, this difference was not statistically significant (95% CI, −0.37 to 4.34; P = 0.097). Therefore, this observation cannot be interpreted as evidence of a treatment-related height gain, particularly in the absence of an untreated control group. Furthermore, the large standard deviation underscores the wide variability in individual growth responses, suggesting that treatment benefit was not uniform across participants and limits the interpretation of the mean difference as evidence of treatment-related height gain. The relatively modest difference between height at final follow-up and baseline PAH observed in our cohort should be interpreted in the context of the considerable heterogeneity in pubertal and skeletal maturity of study participants. The mean bone-age advancement was 2.24 ± 0.9 years, and 14.6% of participants had already experienced menarche before GnRHa initiation. Among these participants, mean age at GnRHa initiation was 8.54 ± 0.71 years and mean bone age was 11.23 ± 1.44 years. Moreover, the mean difference between height at final follow-up and baseline PAH in this subgroup was only 0.10 ± 5.20 cm, indicating that attained height was, on average, close to the baseline predicted adult height. These findings suggest that some participants may have had relatively limited residual growth potential at treatment initiation, which may have contributed to the modest overall height benefit observed in this cohort compared with others in which treatment was initiated at earlier stages of pubertal development (2, 3). Our results align with those of a previously reported cohort from the same center which demonstrated a mean final adult height and a height gain of 158.5 cm and 2.9 cm respectively (16). Height at final follow-up observed in our cohort falls within the range reported in other longitudinal studies and systemic meta-analysis that included 98 studies, in which the mean final height among treated girls ranged between 153 and 160.5 cm (4, 21–23). A recent meta-analysis of 15 studies demonstrated that girls receiving GnRHa have significantly greater final adult height and height gain compared to untreated controls, with a weighted mean difference (WMD) in final adult height and height gain of 3.18 cm and 2.52 cm respectively (24). Moreover, evidence from a systematic review of 29 studies has shown that the gain in final height among patients treated with GnRHa ranges between 2 and 10.5 cm (3). These variable individual responses across studies, as well as the wide standard deviation in height gain observed in our cohort (10.1 cm), are likely attributable to differences in age at treatment initiation, the degree of bone age advancement at diagnosis, the underlying etiology of precocious puberty, the duration of therapy, and adherence to treatment (4). Moreover, using either the average or accelerated Bayley-Pinneau tables for PAH may partially explain variability in individual responses (3). In our cohort, height at final follow-up was significantly associated with GnRHa treatment duration. This association should be interpreted with caution as the apparent benefit of longer treatment duration likely reflects earlier diagnosis and earlier treatment initiation, which are well-established predictors of greater adult height outcomes in children with CPP (2, 3, 21). On the other hand, the negative association between height at final follow-up and bone age at diagnosis may reflect a limited remaining growth potential with late diagnosis and more advanced bone age at treatment initiation (2, 3, 16, 21). Participants who had experienced menarche before GnRHa initiation received a shorter mean treatment duration than the overall cohort (1.91 ± 0.80 vs. 3.14 ± 1.90 years), and their mean height at final follow-up and baseline PAH difference was only 0.10 ± 5.20 cm. These findings suggest that pubertal and skeletal maturity at treatment initiation, rather than treatment duration alone, may be important determinants of the observed height outcomes. Ultimately, accumulating evidence emphasizes that GnRHa therapy in girls with precocious puberty arrests premature bone maturation and may be associated with favorable adult height outcomes, particularly when initiated in a timely manner.

In this cohort, almost 48% of girls were obese and overweight at the initiation of GnRHa therapy, aligning with other several studies (4, 20, 22, 25). These findings underscore the possibilities that early activation of hypothalamic–pituitary–gonadal axis may play a role in the development of obesity in girls with CPP as well as the influence of baseline adiposity on this early activation (11). Among our study participants, BMI SDS remained stable during GnRHa therapy and between CPP diagnosis and long-term follow-up, with significant improvement after treatment discontinuation. Our findings were consistent with several longitudinal studies and a recent meta-analysis which concluded that while temporary BMI changes may occur during GnRHa therapy, long-term weight outcomes are generally favorable and BMI tends to return to pretreatment levels by adult height (5, 22, 25, 26). Longitudinal assessment of BMI categories among our cohort, revealed that most normal-weight children largely maintained their BMI, while overweight participants were at higher risk of progressing to obesity. Despite these individual shifts, there was no statistically significant overall change in the distribution of BMI categories from baseline to final follow-up (χ² = 4.05, df = 3, P = 0.256). These findings indicate that BMI changes may be more closely related to baseline BMI and early activation of hypothalamic–pituitary–gonadal axis than GnRHa therapy. Also, they highlight the importance of monitoring overweight children closely during treatment and early implementation of appropriate lifestyle interventions to prevent progression to obesity.

Insulin resistance has been associated with central precocious puberty, and hyperinsulinemia may promote early activation of the hypothalamic-pituitary-gonadal axis, potentially influencing pubertal timing and metabolic risk (11–13). However, there are limited studies on metabolic outcomes in patients with CPP treated with GnRHa (12, 14). Because Age and population-specific thresholds for HOMA-IR and QUICKI were not available for our younger participants, we did not estimate the prevalence of insulin resistance in the overall cohort. Among participants aged ≥18 years, 50% met the prespecified adult-derived threshold for HOMA-IR and 50% met the corresponding threshold for QUICKI. These findings should be interpreted with caution, as the observed proportions depend on the specific adult-derived cutoff values applied (17). The high prevalence of overweight and obesity (54.9%) in this cohort at final evaluation may also have contributed to this finding as excess adiposity is a strong predictor of insulin resistance. Despite the absence of a control group for comparison, insulin sensitivity indices were significantly associated with BMI or BMI-SDS across different assessment periods, whereas no association was observed with GnRHa treatment duration. These findings suggest an association between adiposity and insulin sensitivity indices in this cohort; however, the relative contributions of underlying predisposition, background population risk, pubertal timing, and GnRHa therapy cannot be determined with the present study design. In line with our results, J. Park et al. clearly demonstrated that GnRHa therapy had minimal impact on insulin resistance in normal-weight girls, but overweight and obese participants showed increased insulin resistance, highlighting the importance of individualized weight management strategies for CPP patients (14). In a historical case-control study of 142 women with CPP aged 20–50 years, diabetes prevalence was low and did not differ between former CPP patients and controls, or between treated and untreated females (22). However, a small study of girls with CPP reported that insulin sensitivity was reduced at diagnosis, with further deterioration of insulin resistance markers observed during GnRHa treatment (12).

While GnRHa therapy effectively suppresses premature activation of the hypothalamic-pituitary-gonadal axis, there is ongoing interest in understanding its potential effects on future fertility, menstrual function, and overall reproductive health. In our cohort, the mean age at menarche was 12.65 years, with spontaneous menarche occurring approximately one year after discontinuation of GnRHa therapy. Fertility was not formally evaluated but three participants reported spontaneous pregnancies resulting in healthy children. Given the small number of pregnancies observed, these findings are descriptive and insufficient to draw conclusions regarding long-term reproductive function or fertility after GnRHa treatment. Guaraldi et al. reported more than 100 pregnancies among women previously treated for CPP, including 97 uneventful pregnancies resulting in healthy children (27). Nevertheless, larger studies with prolonged follow-up are needed to better characterize reproductive outcomes in this population.

Diagnosing polycystic ovary syndrome (PCOS) in younger participants remains inherently challenging because several features of normal pubertal development may overlap with PCOS manifestations. Therefore, in this study, participants with gynecological age < 8 years were diagnosed using the 2023 International Evidence-Based Guideline for the Assessment and Management of Polycystic Ovary Syndrome and the international adolescent-specific recommendations, which require persistent ovulatory dysfunction and clinical and/or biochemical hyperandrogenism, while polycystic ovarian morphology was not considered a diagnostic criterion because it is common during normal puberty (19, 20, 28).

The development of polycystic ovary syndrome (PCOS) remains a debated outcome in women with a history of CPP. Among our study participants, 22.4% of participants met the diagnostic criteria for PCOS. This finding is higher than general population estimates in women of Middle Eastern origin, which range from 8.9–11.9% (29). Population-based data on the prevalence of PCOS in Jordan are limited. However, estimates from the Global Burden of Disease 2019 study indicate that the age-standardized incidence rate of PCOS in Jordan has increased from 57.56 per 100,000 women in 1990 to 70.60 per 100,000 in 2019, corresponding to approximately 2,359 new cases in 2019 (30). These estimates may provide useful insight into the local epidemiological burden of PCOS; however, they are not directly comparable with our findings. This observed frequency of PCOS should be interpreted with caution because of the selected nature of the cohort, the small number of adult participants, the possibility of participation bias due to substantial loss to follow-up, and the overlap between PCOS diagnostic features and normal pubertal development in adolescents and young women. Nevertheless, this finding may suggest a potentially increased susceptibility to PCOS among women with a history of CPP (8). This observed frequency is comparable to most previously reported studies, which ranged from 2–26% in treated CPP patients (5, 10, 25, 31, 32), but lower than rates observed in studies such as Franceschi et al. and Chiavaroli et al. (33, 34). These discrepancies could be related to heterogeneity in study design, PCOS diagnostic criteria, as well as variation in gynecological age, follow-up duration, and obesity prevalence among study populations. Furthermore, variation in participant selection and referral patterns may contribute to differences in the reported PCOS prevalence across studies.

Differences in menstrual irregularity, hyperandrogenic features, and PCOM between the definite-PCOS and no-definite-PCOS groups are expected because these features contributed to PCOS classification (19, 20). Although P values could be reconstructed from the reported counts and are included for completeness, they are not used as independent evidence of diagnostic separation. The exploratory comparisons of characteristics not used to define PCOS did not identify clear group differences, but the small number of definite PCOS cases limits precision.

The variability in pubertal and skeletal maturity at GnRHa initiation indicates substantial clinical heterogeneity. A multivariable model of factors associated with PCOS was not retained because only 15 definite PCOS cases were available and the proposed model would have been unstable. Consequently, the observed PCOS frequency should be considered descriptive rather than evidence of a causal association with GnRHa therapy. Prior studies have also reported inconsistent findings regarding whether later PCOS reflects treatment, the underlying CPP phenotype, adiposity, or shared neuroendocrine susceptibility (5, 10, 20, 31–35). Among participants who completed PCOS assessment, 40.3% were classified as at risk, supporting longitudinal reassessment in those who have not yet met definite criteria.

At long-term follow-up, the cohort’s mean lumbar and femoral BMD Z-scores were within age- and sex-referenced ranges, in line with Alessandri et al., who reported BMD values comparable to controls three years after GnRH agonist therapy (36). BMD was measured only once after treatment, without pretreatment measurements or an untreated comparison group; therefore, the study cannot determine longitudinal bone accrual or whether GnRHa affected skeletal outcomes. Differences in BMD across BMI categories may reflect body size, mechanical loading, and adipose-derived hormonal influences rather than prior treatment (37). The BMD findings should therefore be interpreted as a cross-sectional description of participants who returned for evaluation.

This is the first study in the region that comprehensively assessed anthropometric, metabolic, reproductive and bone mineral density outcomes in the same cohort of patients with CPP almost seven years after discontinuation of GnRHa. The use of the recent International Evidence-Based Guideline for the Assessment and Management of Polycystic Ovary Syndrome and the international adolescent-specific recommendations will add to the strength of this study.

The results should be interpreted in light of several important limitations. First, only 82 of 265 eligible patients (30.9%) completed long-term assessment; 145 could not be reached and 38 declined. Baseline information was incomplete for many nonparticipants, precluding a reliable participant-versus-nonparticipant comparison. In addition, participants were recalled several years after treatment discontinuation, and the decision to participate may have been influenced by ongoing health concerns, such as PCOS-related features or insulin resistance. Selection bias may therefore be substantial, and the observed frequencies of PCOS-related, metabolic, and skeletal outcomes may not represent the full eligible cohort. The absence of an untreated or population-based control group further limits the ability to distinguish findings potentially related to GnRHa treatment from those reflecting background population risk. Second, the cohort was clinically heterogeneous: 11 participants had breast Tanner stage 1 recorded at initial evaluation despite biochemical central HPG-axis activation, and 12 had experienced menarche before treatment. Although exclusion of the Tanner-stage-1 subgroup did not materially change principal descriptive findings, residual heterogeneity remains. Third, retrospective records were incomplete for age at pubertal onset, Tanner stage at treatment initiation, diagnosis-to-treatment interval, and adherence. Fourth, missing outcome data were handled by available-case analysis without imputation, resulting in different denominators and potential additional selection bias. Fifth, final follow-up included both adolescents and adults; longitudinal BMI category and BMI-SDS comparisons across this age span should be interpreted cautiously. HOMA-IR and QUICKI thresholds were adult-derived and were therefore applied categorically only to adults. Finally, the height regression is observational, PCOS comparisons were limited by only 15 cases, multiple exploratory tests were performed without multiplicity adjustment, and BMD was measured only cross-sectionally. These limitations restrict causal inference and generalizability.

In conclusion, among the 30.9% of eligible patients who completed long-term evaluation, attained height was comparable at the group level with baseline predicted adult height and mid-parental height, while mean lumbar and femoral BMD Z-scores were within age- and sex-referenced ranges. PCOS and PCOS-related features were observed, and insulin sensitivity indices were associated with adiposity. These findings support continued metabolic and reproductive follow-up but, because of substantial nonparticipation, cohort heterogeneity, missing outcome data, and the absence of an untreated comparison group, they should be interpreted as descriptive outcomes among evaluated GnRHa-treated participants rather than evidence of treatment effects.

## Data Availability

The raw data supporting the conclusions of this article will be made available by the authors, without undue reservation.
