# Supplementary material for: Long-term clinical outcomes in females with precocious puberty treated with gonadotropin-releasing hormone analogues
Source: Front Endocrinol (Lausanne). 2026 Oct 5;17:1911743. doi: 10.3389/fendo.2026.1911743 (PMC13677348; doi:10.3389/fendo.2026.1911743)
Supplement: Supplementary file 1 [file Table1.docx]

**Supplementary Table S1. Sensitivity analysis comparing principal outcomes in the full cohort and the restricted conventional CPP cohort**

| **Outcome** | **Full cohort (n = 82)** | **Restricted conventional CPP cohort (n = 71)** |
| --- | --- | --- |
| Age at final evaluation, years | 17.61 ± 4.30 | 17.88 ± 4.47 |
| Duration of GnRHa treatment, years | 3.14 ± 1.90 | 3.28 ± 2.02 |
| Age at GnRHa discontinuation, years | 11.63 ± 1.3 | 11.60 ± 1.36 |
| Interval from GnRHa discontinuation to follow-up, years | 6.60 ± 3.80 | 6.83 ± 3.94 |
| Height at final follow up, cm | 159.85 ± 6.50 | 159.99 ± 5.43 |
| Final follow-up height - PAH, cm | 1.98 ± 10.16 | 1.98 ± 10.31 |
| HOMA-IR | 3.76 ± 1.82 | 3.80 ± 1.87 (n = 63) |
| QUICKI | 0.319 ± 0.025 | 0.319 ± 0.024 (n = 64) |
| PCOS, n/N (%) | 15/67 (22.4%) | 13/57 (22.8%) |
| Menstrual irregularity, n/N (%) | 14/67 (20.9%) | 12/57 (21.1%) |
| Interval from treatment discontinuation to spontaneous menarche, years | 1.08 ± 0.90 | 1.12 ± 0.96 |
| Lumbar BMD at long-term follow-up (g/cm²) | 1.06 ± 0.18 | 1.07 ± 0.18 |
| Femoral BMD at long-term follow-up (g/cm²) | 0.94 ± 0.12 | 0.94 ± 0.12 |
